# Using a Recently Developed Self-Report Instrument to Assess Social Anxiety Life Interference in Individuals with Co-occurring Depression: A Known-Groups Analysis

**DOI:** 10.21767/2472-5048.100016

**Published:** 2016-07-11

**Authors:** Antonio F. Garcia, Melina Acosta, Augustine Osman

**Affiliations:** Department of Psychology, The University of Texas at San Antonio, USA

**Keywords:** Social anxiety, Depression, Measurement, Logistic regression, Receiver operator characteristics

## Abstract

Social anxiety is a common condition that often entails substantial adverse impacts on social, educational, and occupational functioning. Moreover, social anxiety often co-occurs with depression, making it difficult to distinguish the unique effects of each condition, which can pose a challenge to effective treatment planning and intervention. Until recently, clinicians have not had access to a validated psychometric instrument that measures the degree of life interference stemming from social anxiety, and that distinguishes life interference associated with social anxiety from that associated with depression. Fortunately, recent work has yielded a novel instrument that combines a measure of social anxiety life interference with a measure of depression life interference, providing a measure that can identify functional disruptions uniquely associated with social anxiety, and that may occur in the presence of comorbid depression. The present article reviews two studies describing the development and psychometric properties of the Social Anxiety and Depression Life Interference Inventory (SADLI-24) and adds to the existing literature by demonstrating the discriminative accuracy of the inventory using a “known-groups” methodology. The article concludes by providing recommendations for the practical application of the SADLI-24 and suggesting future directions for research with the instrument.

## Introduction

Social anxiety disorder (SAD) is a common mental health condition with adverse impacts on social, educational, and occupational functioning. SAD is often comorbid with depression. For instance, 30 to 40% of adolescents with SAD also meet criteria for depression [1]. Unsurprisingly, individuals with SAD have also been found to report low life satisfaction and significant disruptions to activities of daily living [2]. SAD affects a substantial proportion of the population. Lifetime prevalence of SAD in the United States is estimated at 14.3%. In any given year, an estimated 7.1% of the U.S. population (approximately 15 million individuals) meet diagnostic criteria for SAD [3]. Individuals may present with SAD as early as mid-adolescence and many continue to endure the negative consequences of the disorder well into adulthood [4,5].

According to current diagnostic criteria, a diagnosis of SAD requires persistent and extreme fear or anxiety involving one or more evaluative social situations, provoking at least six consecutive months of related anxiety and avoidant behavior, as per the Diagnostic and Statistical Manual of Mental Disorders (DSM-5) [6]. DSM-5 criteria also require that individuals seeking treatment for SAD demonstrate considerable life interference across several functional domains. Unfortunately, self-report measures of life interference stemming from social anxiety have not been readily available for clinical use.

Moreover, a recent literature review evaluating the psychometric properties of existing self-report measures of SAD concluded that none of the reviewed instruments met basic psychometric standards [7]. Upon identifying the need for a psychometrically strong measure of social anxiety life interference, Osman and colleagues developed the Social Anxiety and Depression Life Interference-24 (SADLI-24) Inventory [8]. Unlike traditional self-report measures, the SADLI-24 includes items measuring the level of life interference caused by SAD, rather than the severity or intensity of social anxiety symptoms. The SADLI-24 also contains a subscale measuring depression life interference, enabling clinicians to use SADLI-24 scores to distinguish depression life interference from life interference attributable to social anxiety. Accordingly, the SADLI-24 is a valuable assessment tool that complements existing self-report measures currently used to assess severity of social anxiety symptoms in the presence of significant co-occurring symptoms of depression.

### Development of the SADLI-24

Osman and colleagues reported on the development procedures and psychometric properties of the SADLI-24 in two publications. In the first the authors described the results of four studies [8]. First, the initial item pool was developed and items were inspected for specificity, clarity, relevancy, and representativeness. Scores on the resulting 30-item scale were then subjected to multiple iterations of exploratory factor analysis, resulting in a two-factor inventory with 24 items. Each factor (Social Anxiety Life Interference [SALI-12] and Depression Life Interference [DLI-12]) was composed of 12 items. Osman et al. were then able to replicate this structure in a confirmatory sample. Furthermore, scores on each subscale demonstrated adequate internal consistency reliability. The authors also conducted receiver operator characteristics (ROCs) curve analysis to derive an empirically supported diagnostic cut-point for the SALI-12 and DLI-12 scale scores. Results showed that raw scores of the 24 items were able to differentiate between clinical-level social anxiety (using scores on the SALI-12 scale) and depressive symptoms (using scores on the DLI-12 scale).

In a follow-up publication, Berzins, Garcia, Acosta, and Osman conducted two additional studies to investigate the factor structure of the inventory [9]. Results of exploratory structural equation modeling and bifactor item response theory analyses replicated the two-factor structure of the SADLI-24 [10,11]. Bivariate correlations and principal-axis factoring analyses further supported the convergent and discriminant validity of scores on the SADLI-24 scales.

### Assessing the Performance of the SADLI-24

For the current article, we used a “known-groups” methodology to evaluate the classification accuracy of the SADLI-24 scale scores. This analysis was facilitated by the publication of the abbreviated Social Phobia and Anxiety Inventory (SPAI-23) [12]. This instrument is used to assess individuals for social anxiety symptoms with a high degree of specificity. The Roberson-Nay et al. criteria operationalize social anxiety as a clearly demarcated construct that is distinct from other forms of anxiety such as generalized anxiety and agoraphobia. As such, the SPAI-23 contains an agoraphobia (SPAI-23 AG) scale in addition to a social anxiety scale (SPAI-23 SA), and the former score can be subtracted from the latter to obtain a “pure” measure of social anxiety (SPAI-23 DIFF). Although a clinical cut-off score for the SPAI-23 has not been established, the instrument can be used to define a “known” group of individuals with high levels of social anxiety. Thus, the purpose of the present study was to further examine the classificatory accuracy of the SADLI-24 scale scores in distinguishing individuals with high levels of social anxiety from those with low levels.

## Method

In order to accomplish this objective, we used a dataset consisting of young adults (N=1,065), aged 18 to 29, and created two groups based on level of social anxiety as measured by the SPAI-23. The criterion for inclusion in the “High” social anxiety group was a SPAI-23 DIFF score ≥ 15, corresponding to values in the top quartile of the sample (n=271), whereas the criterion for inclusion in the “Low” social anxiety group was a SPAI-23 DIFF score ≤ 5, corresponding to values in the bottom quartile (n=267). The combined subsample consisted of N = 538 individuals (M age=22.75 [SD=7.28]; 33.5% male / 66.5% female). The Pearson correlation between SALI-12 and DLI-12 scale scores was r=0.671, reflecting substantial shared variance between social anxiety and depression life interference within the sample. Group membership was regressed on the SALI-12 score, with the DLI-12 score included as a covariate to adjust for the effect of any co-occurring depression life interference, thus obtaining a cleaner estimate of the classificatory accuracy of the SADLI-24 as a “pure” measure of social anxiety life interference. Logistic regression was conducted using PROC LOGISTIC within SAS Studio 3.5, with standard plots and outputs. In order to obtain more readily interpretable parameter estimates and effect sizes, independent variables were mean centred and standardized prior to analysis.

## Results

**Figure 1:**
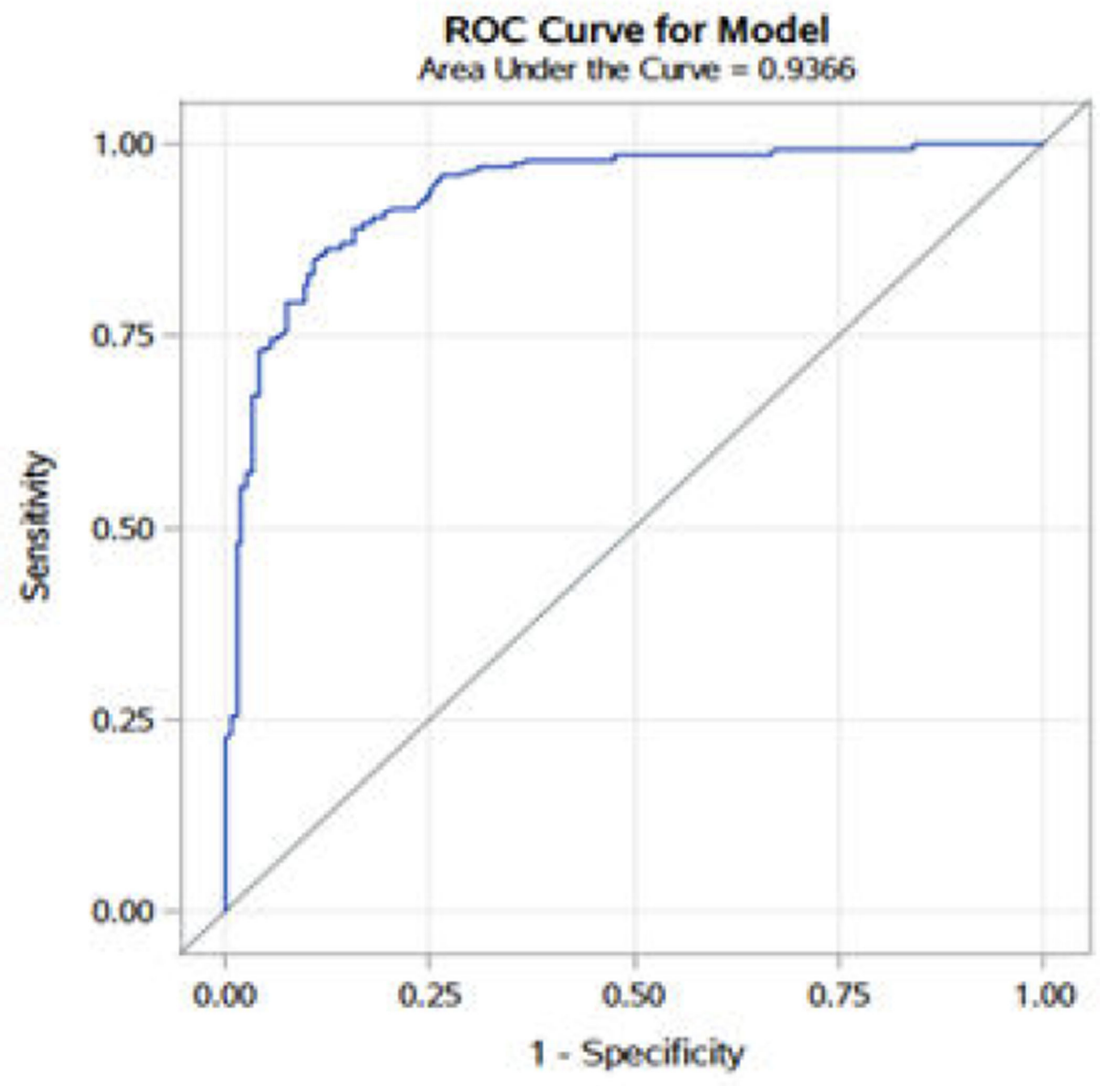
Receiver operator characteristics curve for logistic regression model.

Parameter estimates and model fit information are shown in Table 1. The likelihood ratio, score, and Wald χ^2^ tests were statistically significant at p<0.001, indicating acceptable overall model fit. The Hosmer-Lemeshow test was nonsignificant (χ^2^=13.237, df=8, p=0.104), indicating that the predicted group classifications did not differ significantly from the observed groupings. Controlling for depression life interference as measured by DLI-12, SALI-12 scores strongly predicted membership in the high- and low-social anxiety groups. The logistic regression coefficient for the SALI-12 scores was sizable and highly significant (β=3.359, χ^2^=128.779, df=1, p< 0.001). Controlling for the DLI-12 score, an increase of one SD in the SALI-12 score increased the odds of being classified in the “High” social anxiety group 28-fold (OR=28.763, 95% CI [16.102, 51.380]). As shown in Table 2: For the current study, sensitivity (or the true positive rate) is the proportion of “High Social Anxiety” individuals that were correctly identified as such, whereas specificity, or the true negative rate, measures the proportion of “Low Social Anxiety” individual that were correctly identified as such. The use of the SADLI-24 scores as a predictor of social anxiety “known-groups” yielded strong estimates (>85%) of sensitivity and specificity as well as low false-positive and false-negative rates (<15%). Figure 1 shows the corresponding receiver operator characteristics (ROCs) curve for the logistic regression model, with an area under the curve (AUC) exceeding 0.90 (AUC=0.937, 95% CI [0.916, 0.957]), indicating excellent accuracy of scores on the SADLI-24 when used to discriminate individuals with high levels of social anxiety from those with low levels of symptoms.

## Conclusion

The current results highlight the importance of measuring life interference when assessing individuals presenting with social anxiety. Our social anxiety life interference measure demonstrated high levels of sensitivity, specificity, and overall discriminative accuracy in identifying individuals with high levels of social anxiety. Indeed, social anxiety entails not only psychological distress and avoidance, but also substantial disruptions to normal social functioning and activities of daily living. High levels of social anxiety are associated with diminished life satisfaction, difficulty maintaining gainful employment, and higher risk for substance dependence [13]. Moreover, the link between social anxiety and depression is well established [14]. The SADLI-24 offers a unique contribution to the measurement and clinical assessment of social anxiety by providing a tool for identifying individuals with high levels of social anxiety, entailing substantial life disruptions, with a high degree of discriminative accuracy. Moreover, the inventory contains a depression life interference scale that can be used to partial out the effects of co-occurring depression on life functioning. As discussed by Berzins and colleagues [9], a practical application of the SADLI-24 would be to use a total inventory score for the instrument, should scale users wish to determine the overall extent of life disruption accompanying social anxiety. In addition, due to the excellent discriminative accuracy of the SALI-12 subscale, users seeking to differentiate life disruptions stemming from social anxiety from those stemming from general distress should utilize the original scoring method, which yields a purified measure of social anxiety life interference.

Limitations of the current study include those generally applicable to cross-sectional designs. In particular, a longitudinal design would allow analysis of responsiveness (i.e., the ability to detect clinically important changes in social anxiety life interference over time), as well as an assessment of the reproducibility of scores. In addition, our study sample consisted of healthy community adults, potentially limiting the generalizability of our findings to clinical populations. Further research is needed to examine the psychometric properties of the SADLI-24 for use with highly symptomatic populations. Other important psychometric properties that remain to be addressed in future research are interpretability of scores, test-retest reliability, and measurement invariance. Finally, further research would be necessary to derive a short form of the inventory. Despite these limitations, the current study supports the discriminative accuracy and criterion validity of the SADLI-24 as a self-report measure of life interference stemming from social anxiety.

## Figures and Tables

**Table 1:** Logistic regression analysis of SADLI-24 scores predicting known groups of young adults with high and low levels of social anxiety (N = 538).

							95% CI OR	
Predictor	β	SE β	Wald χ^2^	*df*	*p*	OR	LL	UL
Constant	0.365	0.144	6.385	1	0.012	–	–	–
SALI-12	3.359	0.296	128.779	1	<0.001	28.763	16.102	51.38
DLI-12	−0.507	0.199	6.482	1	0.011	0.602	0.408	0.89
Test			χ^2^		*df*		*p*	
Overall model evaluation								
Likelihood ratio test			397.985		2		<0.001	
Score test			289.24		2		<0.001	
Wald test			154.566		2		<0.001	
Goodness-of-fit test								
Hosmer & Lemeshow			13.237		8		0.104	

Notes: OR=Odds Ratio; SALI-12=Social Anxiety Life Interference-12; DLI-12=Depression Life Interference-12.

**Table 2:** Observed and predicted classifications for high and low social anxiety groups by logistic regression model.

	Predicted		
Observed	High	Low	% Correct
High	234	33	87.64
Low	39	232	85.61
Overall % correct			86.62

Notes: Sensitivity=87.64%; Specificity=85.61%; False positive rate=14.02%; False negative rate=12.45%.
